# OsEDS1 and OsPAD4 Are Involved in Brown Planthopper Resistance in Rice

**DOI:** 10.3390/plants14111612

**Published:** 2025-05-25

**Authors:** Linzhi Fang, Rong Su, Cunyan Li, Xiaodong Liu, Yuanyuan Song, Rensen Zeng, Qiongli Wang, Haitao Cui, Daoqian Chen

**Affiliations:** 1 Key Laboratory of Ministry of Education for Genetics, State Key Laboratory of Agricultural and Forestry Biosecurity, Breeding and Multiple Utilization of Crops, College of Agriculture, Fujian Agriculture and Forestry University, Fuzhou 350002, China; amazingseed@126.com (L.F.); outty22023@outlook.com (R.S.); 15987282757@163.com (C.L.); 17352015063@163.com (X.L.); yyuansong@fafu.edu.cn (Y.S.); rszeng@fafu.edu.cn (R.Z.); 2 Fujian Provincial University Key Laboratory of Crop Biotechnology, Key Laboratory of Ministry of Agriculture and Rural Affairs of Biological Breeding for Fujian and Taiwan Crops, Fujian Agriculture and Forestry University, Fuzhou 350002, China; 3 College of Plant Protection, Shandong Agricultural University, Taian 271000, China

**Keywords:** OsPAD4, OsEDS1, *Oryza sativa*, brown planthopper

## Abstract

The crucial roles of the lipase-like protein enhanced disease susceptibility 1 (EDS1) and phytoalexin deficient 4 (PAD4) in disease resistance in *Arabidopsis* have been identified. However, their function in rice (*Oryza sativa* L.) resistance to brown planthopper (BPH, *Nilaparvata lugens* Stål), the most notorious pest of rice, remains unknown. In this study, the transcript levels of *OsEDS1* and *OsPAD4* were rapidly altered by BPH infestation. Mutation in either *OsPAD4* or *OsEDS1* resulted in increased rice susceptibility to BPH, which was associated with increased honeydew excretion and an increased host preference of BPH. Furthermore, mutation in either *OsPAD4* or *OsEDS1* led to decreased basal levels of salicylic acid (SA) and jasmonic acid (JA) in the absence of BPH, along with the depressed expression of the defense-responsive genes *OsPAL*, *OsICS1*, *OsPR1a*, *OsLOX1*, *OsAOS1* and *OsJAZ11* involved in SA and JA biosynthesis and signaling. The BPH infestation-mediated elevation of SA levels and the expression of SA biosynthesis and signaling genes was dampened in *eds1* and *pad4* plants, whereas BPH infestation-mediated depressions of JA levels and the expression of JA biosynthesis and signaling genes were reversed in *eds1* and *pad4* plants. Taken together, our findings indicated that both OsPAD4 and OsEDS1 positively regulate rice resistance to BPH.

## 1. Introduction

Insect pests impost serious impacts on the production of rice (*Oryza sativa* L.) [1,2], a primarily staple food crop for half of the world’s population. The brown planthopper (BPH) (*Nilaparvata lugens* Stål) is the main insect pest in rice production throughout Asia [1]. As BPH is a rice-specific piercing-and-sucking herbivore, BPH attack not only results in severe damage, ‘hopperburn’, but also transmits rice viruses [3,4]. To date, the mainstay for crop protection against BPH remains chemical insecticides, which has resulted in insecticide resistance, environmental toxicity and concerns for human health [5]. Hence, it is particularly urgent to elucidate the mechanisms underlying rice–BPH interactions and develop resistant rice varieties to this notorious pest.

During millions of years of plant and insect co-evolution, plants have developed intricate defense systems against insect herbivores mediated by multiple signaling pathways, including the plant hormone jasmonic acid (JA) and salicylic acid (SA) signaling pathways [5]. JA is generally considered to play a major role in response to chewing herbivores, while SA is a critical mediator of plant defense responses against sucking herbivores [6,7,8]. In the case of rice–BPH interactions, SA is widely recognized as the key component of rice defenses against BPH. BPH infestation elevates SA levels and gene expression related to SA biosynthesis, particularly in resistant varieties [9,10,11,12]. The expression of *NahG*, which encodes the bacterial salicylate hydroxylase eliminating SA accumulation, decreases rice resistance to BPH [13]. The knockdown of SA biosynthesis genes encoding phenylalanine ammonia lyase (PAL) leads to sensitivity to BPH, while the overexpression of *OsPAL6* and *OsPAL8* enhances rice resistance to BPH [13]. Moreover, the SA pathway is activated after BPH infestation in BPH14-, BPH29- and BPH9-mediated resistance [14,15,16]. On the other hand, several studies indicate that the JA pathway also plays an important role in rice defenses against BPH, particularly in susceptible varieties [17,18]. These observations suggest that the roles of these defense hormones in rice defense against BPH are complicated and depend on the rice genotype.

The functions of the lipase-like protein enhanced disease susceptibility 1 (EDS1) and phytoalexin deficient 4 (PAD4) as key immune activators in plant immunity have been extensively studied [19,20,21,22,23,24,25,26]. In *Arabidopsis thaliana*, AtEDS1 and AtPAD4 function together to confer both basal immunity and effector-triggered immunity (ETI) by stimulating the production of SA and antimicrobial molecules during plant–pathogen interaction [24,27]. It is recognized that activated TNL receptors (intracellular nucleotide-binding leucine-rich repeat receptors with N-terminal Toll–interleukin 1 receptor domains) stimulate AtEDS1-PAD4 basal immunity activity to transcriptionally boost SA levels and repress JA pathways in *Arabidopsis* [24,26]. Moreover, AtPAD4, independently of AtEDS1, functions in resistance to the phloem sap-feeding green peach aphid (*Myzus persicae* Sülzer) [28,29]. Further studies reveal that AtPAD4-mediated defense against green peach aphids is independent of SA or camalexin production [30]. The AtPAD4 lipase-like domain is sufficient for *Arabidopsis* aphid resistance but not for the AtEDS1-dependent activation of basal and effector-triggered pathogen immunity [31]. These results suggest that AtEDS1-PAD4 appears to regulate *Arabidopsis* resistance against pathogens and aphids in a distinct manner. Notably, both OsEDS1 and OsPAD4 also play positive roles in rice resistance to pathogens, whereas they mediate rice resistance against *Xanthomonas oryzae* in a JA-dependent, but not SA-dependent, manner compared with their orthologs in *Arabidopsis* [22,23]. Recently, it was unveiled that the OsEDS1-OsPAD4-OsADR1 immune complex controls rice immune homeostasis and multipathogen resistance with a fundamentally conserved immune-triggering and signaling mechanism in *Arabidopsis* and rice [32,33]. However, whether and how OsEDS1 and OsPAD4 are involved in the rice–BPH interaction remain unknown.

Previous studies indicate that the expression patterns of *OsEDS1* and *OsPAD4* are altered by BPH infestation and that they display differential expression in resistant and susceptible rice varieties during BPH attack, implying the potential involvement of OsPAD4 and OsEDS1 in rice resistance against BPH [9,10,11,12]. Here, *OsPAD4*- and *OsEDS1*- knockout plants were generated to examine the roles of these immune proteins in rice–BPH interactions. Our results reveal that both OsPAD4 and OsEDS1 positively regulate rice defense response against BPH, possibly by modulating the SA and JA signaling pathways.

## 2. Results

### 2.1. BPH Infestation Influences Expression Levels of OsEDS1 and OsPAD4

**Figure 1:**
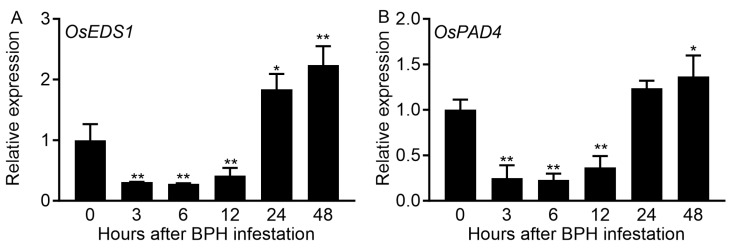
Transcript levels of *OsEDS1* (**A**) and *OsPAD4* (**B**) in leaf sheaths of ZH11 plants after BPH infestation. Thirty-day-old rice seedlings were individually infested with 10 third-instar BPH nymphs for 2 days. Data are represented as means ± SE (*n* = 3). Asterisks above bars indicate significant differences in transcript levels between treatments and controls (* *p* < 0.05; ** *p* < 0.01, one-way ANOVA with LSD post hoc test).

To determine whether *OsEDS1* and *OsPAD4* are involved in rice response to BPH infestation, the transcript levels of these two genes in leaf sheaths infested by BPH were monitored by qRT-PCR. The expression levels of both *OsEDS1* and *OsPAD4* decreased at 3–12 h, then slightly increased at 24 and 48 h after BPH infestation (Figure 1).

### 2.2. Mutations in OsPAD4 and OsEDS1 Decrease Rice Resistance to BPH

**Figure 2:**
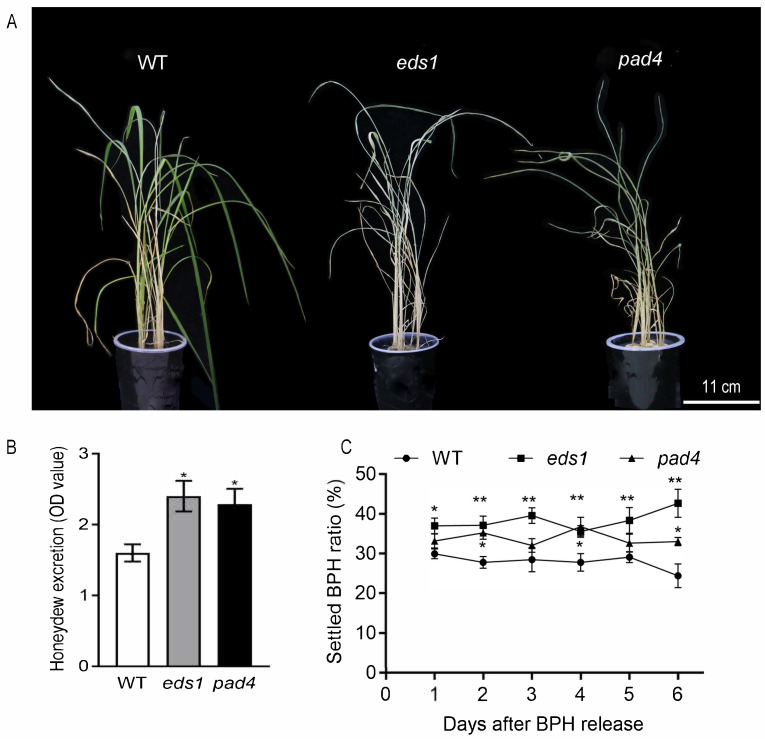
Mutations in *OsPAD4* and *OsEDS1* increased rice susceptibility to BPH. (**A**) Phenotype of *eds1*, *pad4* and ZH11 plants after 7 days of BPH infestation. (**B**) Honeydew excretions (OD value) of BPH insects feeding on *eds1*, *pad4* and ZH11 plants for 2 days. (**C**) BPH settled ratios of host choice assay on *eds1*, *pad4* and ZH11 plants. Data are represented as means ± SE (*n* = 10). Asterisks above bars indicate significant differences between mutants and ZH11 plants (* *p* < 0.05; ** *p* < 0.01, one-way ANOVA with LSD post hoc test).

To further investigate the roles of OsEDS1 and OsPAD4 in rice–BPH interactions, *OsEDS1* and *OsPAD4* knockout rice lines were generated by CRISPR-Cas9 technology inducing a 32 bp deletion at the 2 site from ‘ATG’ (*eds1*) and a ‘T’ insertion at the 452 site from ‘ATG’ (*pad4*), respectively, in the ZH11 background (Figure S1A). Both *eds1* and *pad4* plants did not exhibit abnormal growth phenotypes except for a slight decrease in plant height under normal conditions (Figure S1B,C). Both *eds1* and *pad4* plants showed significant increased susceptibility to BPH infestation. After 7 days of BPH feeding, both *eds1* and *pad4* plants were more severely damaged than ZH11 plants (Figure 2A). Honeydew excretion, a solid indicator of BPH feeding and hence host suitability, was 50.00% and 43.12% higher for the insects feeding on *eds1* and *pad4* plants, respectively, relative to those on ZH11 plants (Figure 2B). Moreover, a host choice behavior test showed that BPH preferred to feed on *eds1* and *pad4* plants compared with ZH11 plants. Among the three genotypes, the settled ratio of BPH nymphs was the highest on the *eds1* plant, the lowest on ZH11 plants and intermediate on the *pad4* plant at almost all time points tested (Figure 2C). However, there was no difference between the three genotypes for the survival rate of BPH after 7 days of infestation (Figure S2).

### 2.3. Mutations in OsEDS1 and OsPAD4 Alter the Expression of Defense-Responsive Genes

**Figure 3:**
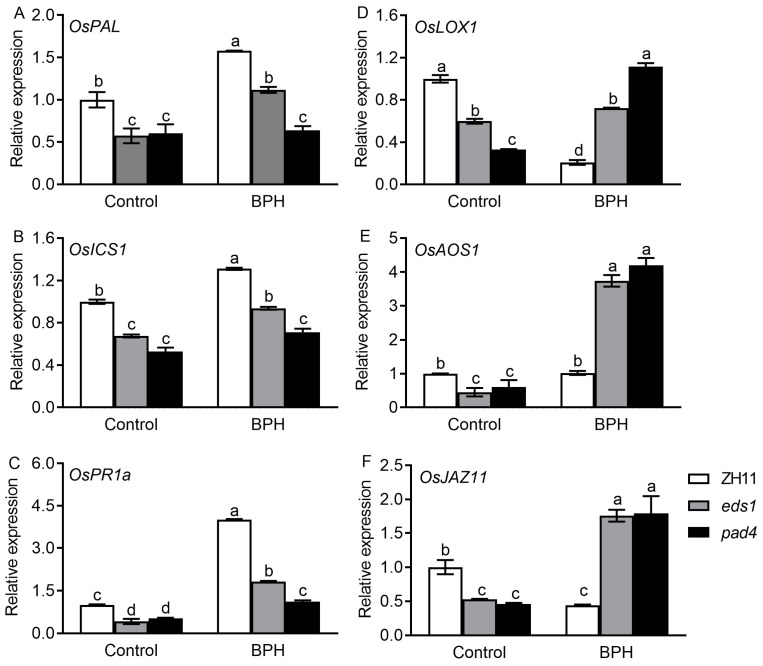
Transcript levels of defense-responsive genes in leaf sheaths of *eds1*, *pad4* and wild-type (ZH11) plants after 2 days of BPH infestation. *OsPAL* (**A**) and *OsICS1* (**B**) are SA biosynthesis genes; *OsPR1a* (**C**) is SA signaling downstream defense-related gene. *OsLOX1* (**D**) and *OsAOS1* (**E**) are JA biosynthesis genes, and *OsJAZ11* (**F**)is JA-responsive marker gene. Data are represented as means ± SE (*n* = 3). Different letters above bars indicate statistically significant differences between treatments (*p* < 0.05, one-way ANOVA with LSD post hoc test).

To investigate how OsEDS1 and OsPAD4 affect rice resistance to BPH, we analyzed the expression levels of defense-responsive genes in ZH11 plants and *OsEDS1* and *OsPAD4* knockout rice lines by qRT-PCR analysis. The transcript of SA biosynthesis genes *OsPAL* (*phenylalanine ammonia-lyase*) and *OsICS1* (*isochorismate synthase 1*) and SA signaling downstream defense-related gene *OsPR1a* (*basic pathogenesis-related gene 1a*) was induced by BPH infestation. However, their transcript levels were all significantly lower in *eds1* and *pad4* plants compared with ZH11 plants under both uninfested (control) and BPH-infested conditions (Figure 3A–C). The transcript levels of JA biosynthesis and signaling genes *OsLOX1* (*lipoxygenase 1*), *OsAOS1* (*allene oxide synthase 1*) and *OsJAZ11* (*jasmonate-zim domain 11*) were significantly depressed in *eds1* and *pad4* plants in the absence of BPH. Their transcript levels were repressed by BPH infestation in ZH11 plants, whereas they were elevated by BPH infestation in *eds1* and *pad4* plants. After BPH infestation, the expression levels of *OsLOX1*, *OsAOS1* and *OsJAZ11* were all significantly higher in *eds1* and *pad4* plants compared with ZH11 plants (Figure 3D,E). 

### 2.4. Mutations of OsEDS1 and OsPAD4 Impair BPH-Induced SA and JA Modulation

**Figure 4:**
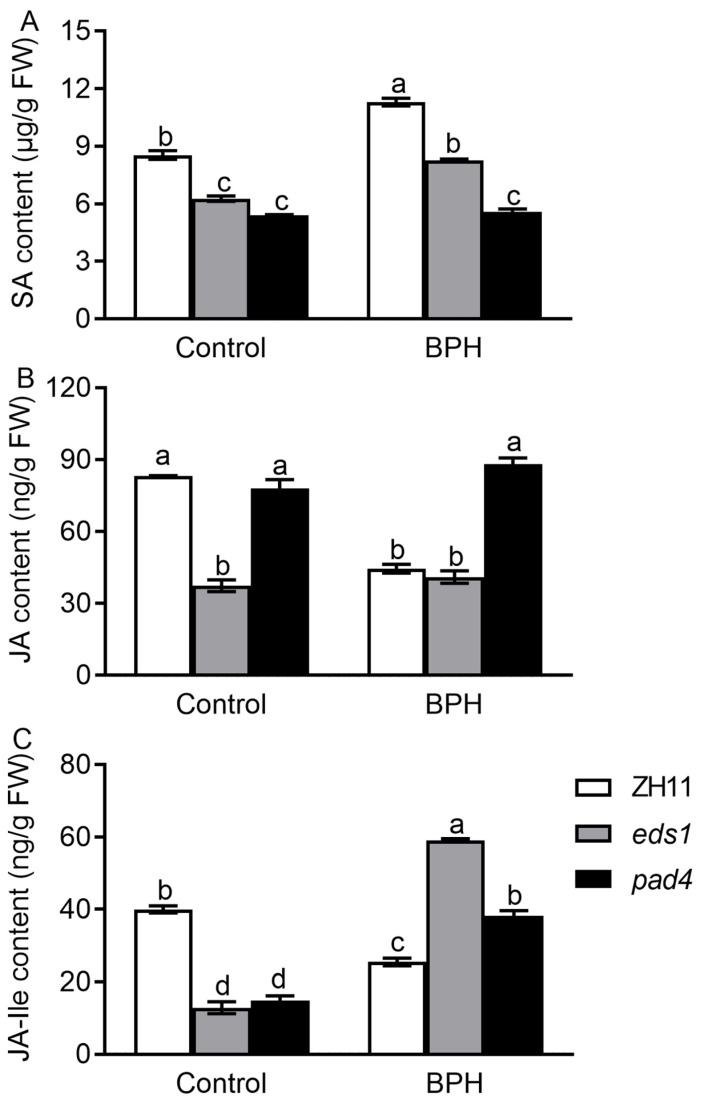
SA (**A**), JA (**B**) and JA-Ile (**C**) levels in leaf sheaths of *eds1*, *pad4* and ZH11 plants after 2 days of BPH infestation. Data are represented as means ± SE (*n* = 3). Different letters above bars indicate statistically significant differences between treatments (*p* < 0.05, one-way ANOVA with LSD post hoc test).

To further confirm the roles of OsEDS1 and OsPAD4 in modulating the SA and JA pathways, we determined the endogenous SA and JA levels in ZH11 plants and *OsEDS1* and *OsPAD4* knockout rice lines after BPH infestation. In line with the expression of defense-responsive genes, SA levels were elevated by BPH infestation, but they were significantly lower in *eds1* and *pad4* plants compared with ZH11 plants under both control and BPH infestation conditions (Figure 4A). In the absence of BPH, *eds1* plants had significantly lower contents of both JA and JA-Ile than ZH11 plants, while *pad4* plants had comparable JA levels but less JA-Ile than ZH11 plants. Both JA and JA-Ile levels were reduced by BPH infestation in ZH11 plants. JA levels remained unchanged in both *eds1* and *pad4* plants after BPH infestation, yet JA-Ile levels were increased by BPH infestation in both *eds1* and *pad4* plants.

## 3. Discussion

EDS1 and PAD4, a pair of sequence-related partners originally characterized in *Arabidopsis*, are two well-known positive regulators in plant immunity. *Arabidopsis* AtEDS1 and AtPAD4 form EDS1-PAD4 complexes to transduce immune signals and promote both pattern-triggered immunity (PTI) and effector-triggered immunity (ETI). Recent studies found that OsEDS1 and OsPAD4, the sequence homologs of AtEDS1 and AtPAD4, respectively, also play crucial positive roles in rice immune homeostasis and multipathogen resistance [33]. It has been frequently reported that BPH infestation also influences the expression levels of *OsEDS1* and *OsPAD4* [9,10,11,12]. However, the roles of OsEDS1 and OsPAD4 in the rice–BPH interaction remain unknown. In this study, the transcript levels of *OsEDS1* and *OsPAD4* in rice sheaths were rapidly responsive to BPH infestation (Figure 1). Mutations in either *OsPAD4* or *OsEDS1* resulted in increased rice susceptibility to BPH, along with increased honeydew excretion and an increased host preference of BPH (Figure 2). These results indicated that OsPAD4 and OsEDS1 are also important positive regulators in rice resistance against BPH, which causes the most serious damage to rice crops among all rice insect pests globally. Further analyses illustrated that impaired BPH resistance was associated with repressed SA signaling and boosted JA signaling in *eds1* and *pad4* mutants (Figure 3 and Figure 4). Therefore, our findings suggested that OsPAD4 and OsEDS1 might regulate rice defense response against BPH by modulating SA and JA signaling.

In *Arabidopsis*, EDS1-PAD4 heterodimer-mediated PTI and ETI defenses involve the transcriptional boosting of the SA pathway and the depression of the JA pathway [20,24,26,34,35]. EDS1-PAD4 not only enhances the expression of the SA biosynthesis gene of *ICS1*, but it also promotes a large number of SA-responsive genes in parallel with SA accumulation [24]. Moreover, EDS1-PAD4 could dampen the JA signaling pathway by suppressing the master transcription factor MYC2, which in turn boosts SA signaling [20]. In rice, OsEDS1 and OsPAD4 are also found to be positive regulators in rice–pathogen interactions, and cryo-electron microscopy structure analysis unveiled the fact that the OsEDS1-OsPAD4-OsADR1 (activated disease resistance 1) immune complex controls rice immune homeostasis and multipathogen resistance, which is a fundamentally conserved immune-triggering and signaling mechanism in *Arabidopsis* and rice [32,33]. In the present study, mutation in either *OsPAD4* or *OsEDS1* dampened BPH infestation-boosted SA signaling, but it reversed BPH infestation-depressed JA signaling (Figure 3 and Figure 4). These findings suggested that OsEDS1 and OsPAD4 might confer rice resistance against BPH in a conserved manner by boosting the SA pathway and dampening the JA pathway in rice–BPH interactions. In addition, it is reported that OsEDS1 and OsPAD4 positively regulate rice immunity against *X. oryzae* in a JA-dependent manner [22,23]. In this study, the depression of the JA signaling pathway in *eds1* and *pad4* mutants was also observed in the absence of BPH, but the JA signaling pathway was fundamentally enhanced by a mutation in either *OsPAD4* or *OsEDS1* after BPH infestation (Figure 3 and Figure 4). Therefore, it is possible that OsEDS1 and OsPAD4 share both similarities and differences with their *Arabidopsis* orthologs in a way to regulate pathogen resistance, and they might function in rice–pathogen interactions and rice–BPH interactions in a distinct manner.

Phytohormones SA and JA are essential for controlling defense signaling in plant resistance against insect pests [5]. It is generally considered that SA and JA play distinct roles in mediating plant antiherbivore defenses against chewing and phloem-feeding insects [8]. However, in the case of rice–BPH interactions, the roles of these defense hormones in rice defense response are complicated and depend on the rice genotype. SA is widely recognized as the key component of rice defenses against BPH, particularly in resistant varieties. It has been found that the SA pathway is boosted in BPH14-, BPH29- and BPH9-mediated defense following BPH feeding [14,15,16]. In contrast, BPH infestation induces the defense associated with the JA pathway in susceptible varieties [17,18]. In the present study, we also found that the SA pathway was activated by BPH infestation in resistant ZH11 plants, whereas the JA pathway was boosted by BPH infestation in susceptible *eds1* and *pad4* mutants (Figure 3 and Figure 4). These results are in accordance with an observation made in the BPH-resistant introgression line RBPH54 possessing a recessive allele of *BPH29* and BPH-susceptible transgenic lines overexpressing a *BPH29* dominant allele [16]. Moreover, higher basal JA contents were observed in susceptible wild-type plants compared with the resistant *oseil1* mutant [36]. And the enhanced JA levels observed are associated with increased susceptibility in the *hpl3-1* mutant [37]. One possible explanation for this might be the classic binary model of JA and SA defense mechanisms, which indicates that these phytohormones play opposing roles in mediating defense responses against chewing and sucking insects [9]. It has been reported that silencing JA biosynthesis or signal transduction genes reduced the levels of JA and TrypPI, thus improving rice leaffolder and striped stem borer larval performance while simultaneously increasing the levels of SA and H_2_O_2_ to enhance (or at least not adversely affect) BPH resistance [17,36,37,38].

## 4. Materials and Methods

### 4.1. Plant Materials and Growth Conditions

The wild-type rice variety ZH11 (*Oryza sativa* L. cv. Zhonghua No. 11) was used in this study. *OsEDS1* and *OsPAD4* knockout mutants were created by CRISPR-Cas9 technology inducing a 32 bp deletion at the 2 site from ‘ATG’ (*eds1*) and a ‘T’ insertion at the 452 site from ‘ATG’ (*pad4*) in the ZH11 background, respectively (Figure S1A). All the rice materials used in this study were propagated for at least 3 generations in disease- and pest-controlled propagation fields under strict phytosanitary management.

### 4.2. Plant Growth Conditions

Seeds of rice were subjected to surface sterilization using 2.5% (*v*/*v*) sodium hypochlorite (NaClO) for 30 min, rinsed five times and then soaked in tap water for two days for germination. After 7 days, uniform healthy seedlings were transplanted into a plastic box (L × W × H: 35 cm × 25 cm × 12 cm) containing 5 L full-strength modified Kimura B nutrient solution (pH: 5.6, renewed every 3 d) as previously described by Chen et al. [39]. Rice plants were cultivated for an additional 21 days in a growth chamber under a 12 h/12 h day–night cycle, with the temperature regime at 27 °C/23 °C and a light intensity of 300 μmol m^−2^ s^−1^.

### 4.3. BPH Rearing and Infestation

BPH colonies were originally collected from rice fields in the campus of Fujian Agriculture and Forestry University in Fuzhou, China, and maintained on plants of Taichung Native 1 (a variety susceptible to BPH) under controlled conditions (as for plants).

For plant sampling, 30-day-old rice seedlings were individually infested with 10 third-instar BPH nymphs, and leaf sheaths were harvested after 0 and 2 days. Sampled leaf sheaths from three rice seedlings were pooled together as a replicate, immediately frozen in liquid nitrogen and stored at −80 °C for further analysis.

For a BPH tolerance bioassay, 30-day-old rice seedlings were transplanted to plastic pots (D × H: 11 cm × 15 cm) containing 1 L nutrient solution (four plants per pot). Rice plants were infested with BPH nymphs (30 third-instar nymphs per plant) and confined in ventilated plastic cylinders (D H: 11 cm, 50 cm) to prevent BPH from escaping. Plant status was observed every day, and plants were photographed after 7 days of infestation.

Honeydew production was measured to determine feeding rates. Rice seedlings were transplanted to plastic pots (one plant per pot). A piece of filter paper (9 cm in diameter) was laid flat on the cup lid, and the rice seedling leaf sheaths were allowed to pass through the filter paper. Each rice plant was inoculated with 10 third-instar female BPH nymphs starved for 2 h in advance and confined in ventilated plastic cylinders. Two days later, filter papers were collected and stained with 0.25% (*w*/*v*) ninhydrin solution. Stained filter paper was eluted with a mixture of 0.8 mL 1.2% copper sulfate and 4.2 mL 85% ethanol. The OD value of the eluent was determined at 570 nm to represent the amino acid content in BPH honeydew, as previously described by Friedman [39]. Each treatment included 10 replicates.

For BPH host choice behavior analysis, each pair of *oseds1*, *ospad4* and ZH11 seedlings was transplanted to plastic pots, inoculated with 40 third-instar BPH nymphs and confined in ventilated plastic cylinders. The number of BPH on each rice seedling was recorded 1, 2, 3, 4, 5 and 6 d after inoculation, and the settled ratio was calculated. Each pair included 10 replicates.

For BPH survival rate analysis, rice seedlings were transplanted to plastic pots (one plant per pot). Each rice plant was inoculated with 15 second-instar nymphs and confined in ventilated plastic cylinders. The number of nymphs alive on each plant was counted daily for 7 days, and the survival rate was calculated. Each treatment included 15 replicates.

### 4.4. Gene Expression Analysis

For gene expression analysis, frozen leaf sheath samples (approximately 0.1 g) were used for RNA extraction following the Eastep® Super Total RNA Extraction kit manual (Shanghai Promega, Shanghai, China). RNA was reverse-transcribed into cDNA using the ReverTra Ace@ qPCR RT master Mix with gDNA remover (TOYOBO, Shanghai, China). RT-qPCR was performed using the 2 × Taq Pro Universal SYBR qPCR Master Mix (Vazyme, Nanjing, China) on an Applied Biosystems Step One Plus system (Applied Biosystems, Foster City, USA) with *OsActin* as the internal reference gene. Primer specificity was validated through primer-BLAST and verified by a melt curve analysis. The 2^−∆∆Ct^ method was used for calculating the relative expression of genes. All experiments were performed in triplicate using three biological replicates per treatment. Gene-specific primers are listed in Table S1.

### 4.5. Quantification of JA, JA-Ile and SA

Frozen leaves were used for the quantification of JA, JA-Ile and SA by UPLC-MS/MS as described by Lu et al. [40]. In brief, a fine powder of leaf sheaths (approximately 150 mg for each sample) was dissolved in 1 mL of ice-cold ethyl acetate spiked with internal standards (20 ng of D_6_-JA, 5 ng of D_5_-JA-Ile, 5 ng of D_4_-SA), and it was vortexed for 10 min. After centrifugation at 16,100× *g* for 10 min at 4 °C, supernatants were transferred to 2 mL tubes for subsequent vacuum evaporation. The residue was resuspended in 0.5 mL of 70% (*v*/*v*) methanol and centrifuged at 16,100× *g* for 10 min at 4 °C. The resulting supernatant was transferred to glass vials and then subjected to HPLC-MS/MS analysis (LCMS-8040, Shimadzu, Kyoto, Japan). The SA, JA and JA-Ile contents were calculated according to a ‘concentration–peak area’ curve of standard samples. Each treatment included three replicates.

### 4.6. Statistical Analysis

Data analyses were carried out by SPSS 22.0 (SPSS Inc., Chicago, IL, USA). For all data, we performed an analysis of variance (ANOVA) followed by a least significant difference (LSD) post hoc test.

## 5. Conclusions

In summary, the present study reveals that OsPAD4 and OsEDS1, two well-known crucial immune activators, are responsive to BPH infestation and involved in rice defense response to BPH by modulating SA and JA signaling. Further studies on the functional characterization of the key components and underlying mechanisms in the EDS1-PAD4 module mediating rice BPH resistance are required for the future development of resistant varieties to control this devastating insect.

## Data Availability

The original contributions presented in this study are included in the article. Further inquiries can be directed to the corresponding authors.

## Supplementary Materials

The following supporting information can be downloaded at https://www.mdpi.com/article/10.3390/plants14111612/s1, Figure S1: Characterization and growth phenotypes of *eds1* and *pad4* mutants; Figure S2: Survival rates of BPH feeding on ZH11 and knockout mutant plants for 7 days, Table S1: Primers used for real-time quantitative PCR.

## Abbreviations

The following abbreviations are used in this manuscript:

BPH	*Nilaparvata lugens* Stål
JA	Jasmonic acid
SA	Salicylic acid
EDS1	Enhanced disease susceptibility 1
PAD4	Phytoalexin deficient 4
*OsPAL*	*Phenylalanine ammonia-lyase*
*OsICS1*	*Isochorismate synthase 1*
*OsPR1a*	*Basic pathogenesis-related gene 1a*
*OsLOX1*	*Lipoxygenase 1*
*OsAOS1*	*Allene oxide synthase 1*
*OsJAZ11*	*Jasmonate-zim domain 11*
