# Supplementary material for: OsEDS1 and OsPAD4 Are Involved in Brown Planthopper Resistance in Rice
Source: Plants (Basel). 2025 May 25;14(11):1612. doi: 10.3390/plants14111612 (PMC12157244; doi:10.3390/plants14111612)
Supplement: Supplementary file 1 [file plants-14-01612-s001.zip › plants-3622431-supplementary.pdf]

# Supplementary Materials

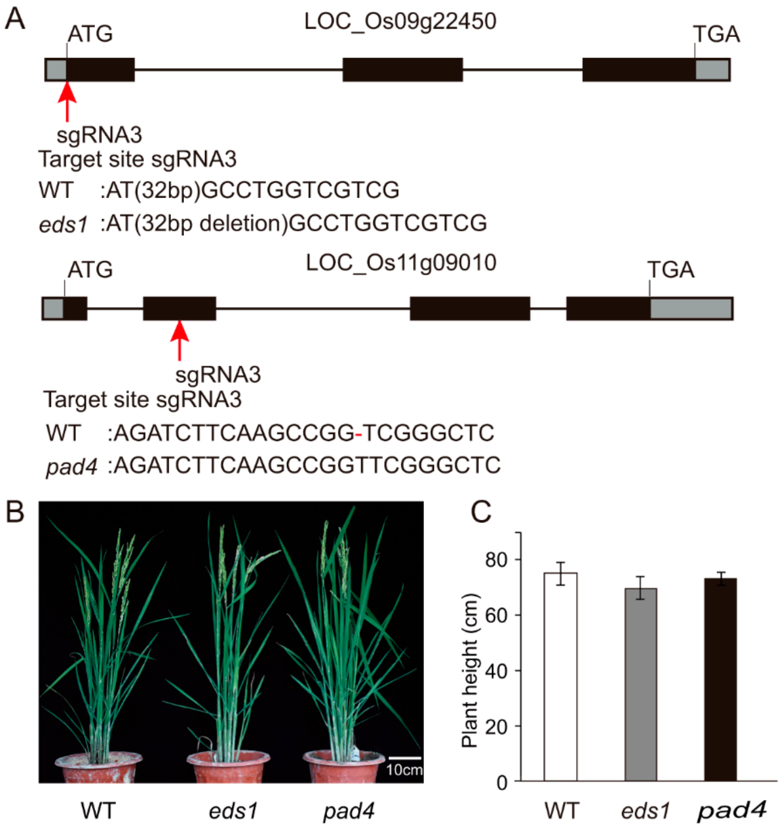


**Figure S1**. Characterizations and growth phenotypes of *eds1* and *pad4* mutants. (A) Characterization of *eds1* and *pad4* mutants. Both *OsEDS1* and *OsPAD4* knockout mutants were created by CRISPR-Cas9 technology. The *OsEDS1* knockout mutant (*eds1*) harbors a 32-bp deletion at 2 site from 'ATG'. The *OsPAD4* knockout mutant (*pad4*) harbors a 'T' insertion at 452 site from 'ATG'. (B) Growth phenotypes of WT and knockout mutant plants. (C) The heights of WT and knockout mutant plants. Data are represented as means ± SE ($n$ = 3).

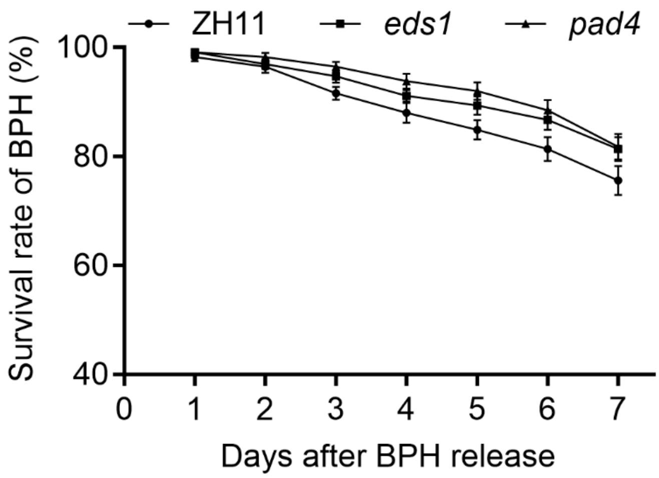


Figure S2. Survival rates of BPH feeding on ZH11 and knockout mutant plants for 7 days. Data are represented as means ± SE ($n$ = 3).

**Table S1**. Primers used for real-time quantitative PCR

| Genes | Primer sequence (5′→3′) |
|---|---|
| *OsPAL* | F: GACTCTGGTGATGGTGTCAGC |
| | R: GGCTGGAAGAGGACCTCAGG |
| *OsICS1* | F: CGTGCTTATGGTGCTATCC |
| | R: TGTCCAAGAAAGTGAATCATCC |
| *OsPR1a* | F: TCGTATGCTATGCTACGTGTTTATG |
| | R: TCGGATTTATTCTCACCAGCA |
| *OsLOX1* | F: GTACGCTGGGTTCACAGCTC |
| | R: TCAGATGGATGTGCTGTTGG |
| *OsAOS1* | F: CGGGACATGGTGGTGGAGA |
| | R: GGAGTCGTATCGGAGGAAGAGC |
| *OsJAZ11* | F: AGTACATGAAGGAGCACAGTG |
| | R: CTTCCTTTCTTGCGTGTCTTTC |
| *OsActin* | F: GACTCTGGTGATGGTGTCAGC |
| | R: GGCTGGAAGAGGACCTCAGG |
